# A New Hybrid Stepper Motor, Compliant Piezoelectric Micro-Tweezer for Extended Stroke

**DOI:** 10.3390/mi14061112

**Published:** 2023-05-25

**Authors:** Ioan Alexandru Ivan, Dan Cristian Noveanu, Valentin Ion Gurgu, Veronica Despa, Simona Noveanu

**Affiliations:** 1 École Nationale d’Ingénieurs de Saint-Étienne (ENISE), LTDS, CNRS UMR 5513, Ecole Centrale de Lyon, 42023 Saint-Etienne, France; ioan-alexandru.ivan@enise.fr; 2 National Institute of Materials Physics (NIMP), Magurele, 077125 Bucharest, Romania; 3 Materials Science and Engineering Department, Technical University of Cluj-Napoca, 400641 Cluj-Napoca, Romania; 4 Institute of Multidisciplinary Research for Science and Technology, Valahia University of Targoviste, 130004 Targoviste, Romania; valentin.gurgu@valahia.ro (V.I.G.); dumiver@yahoo.com (V.D.); 5 Mechatronics and Machine Dynamics Department, Technical University of Cluj-Napoca, 400641 Cluj-Napoca, Romania; simona.noveanu@mdm.utcluj.ro

**Keywords:** microgripper, micromanipulation, compliant mechanism

## Abstract

The revolutionary economic potential of micro and nanotechnology is already recognized. Micro and nano-scale technologies that use electrical, magnetic, optical, mechanical, and thermal phenomena separately or in combination are either already in the industrial phase or approaching it. The products of micro and nanotechnology are made of small quantities of material but have high functionality and added value. This paper presents such a product: a system with micro-tweezers for biomedical applications—a micromanipulator with optimized constructive characteristics, including optimal centering, consumption, and minimum size, for handling micro-particles and constructive micro components. The advantage of the proposed structure consists mainly in obtaining a large working area combined with a good working resolution due to the double actuation principle: electromagnetic and piezoelectric.

## 1. Introduction

In the last two decades, research in the field of micromanipulation has been developed and intensified, especially due to an increased demand for these microelectromechanical systems (MEMS) with frequent use in medicine, microelectronics, or biotechnology, fields in which objects with dimensions 1–100 µm are precisely handled.

The specialized literature reports a multitude of actuation principles for micro-robotics, among which we mention: piezoelectric [1,2,3], thermal [4,5], electrostatic [6,7], electromagnetic [8,9], and shape memory alloys [10,11]. The range of unconventional drives is much wider and constantly expanding during development, often due to the adoption of non-conformist solutions for obtaining a mechatronic product [12]. Of these, the usual commercial, technical solutions for micro-tweezers are based on easy-to-manufacture integrated electrostatic micro-actuators (MEMS), which, however, have the disadvantage of mechanical fragility.

Thanks to the improvements in technology, microgrippers can be realized in a monolithic way, such as a compliant mechanism with a flexure hinge with different shapes like corners filleted with different radii, circular, elliptic, parabolic, etc. [13,14]. Compliant mechanisms offer several benefits over traditional rigid mechanisms. They can be lighter and less expensive, and they can also exhibit better energy absorption, reduced stress concentration, and improved reliability. They are also more tolerant of misalignment and can be used to achieve more complex motions.

Examples of compliant mechanisms include flexures, leaf springs, and bellows. Flexures are thin beams or plates designed to bend in a specific direction and are often used as hinges or suspension elements [15].

Compliant microgrippers are a type of robotic gripper designed to be flexible and compliant, allowing them to conform to a wide range of objects and surfaces. They are constructed using flexible materials that can deform under stress and have a higher degree of compliance than traditional grippers, which can benefit certain human–robot interaction contexts. These grippers have become increasingly popular in various industries, from manufacturing to healthcare, due to their numerous advantages over traditional rigid grippers. The key future of compliant grippers is their ability to deform and conform to the shape of the object they are gripping, which can make them safer and more efficient in certain applications. One of the most significant advantages of compliant microgrippers is their versatility. Unlike traditional rigid grippers, which are designed for specific shapes and sizes of objects, compliant microgrippers can conform to a wide range of shapes and sizes. This makes them ideal for applications where objects vary in shape or size, such as in medical procedures or manufacturing processes. Compliant microgrippers are also known for their precision. Because they are flexible and compliant, they can grip objects with a high degree of accuracy and precision. This is particularly important in applications where precision is critical, such as in micro-assembly or medical procedures. Another advantage of compliant microgrippers is their safety. Traditional rigid grippers can be dangerous when used near humans, as they can cause injury if they come into contact with a person. Compliant microgrippers, on the other hand, are much safer to use, as they are designed to be flexible and compliant. This makes them ideal for use in applications where human interaction is required, such as in medical procedures or collaborative robotics. Compliant microgrippers are also more efficient than traditional rigid grippers. Because they are designed to conform to the object being gripped, they require less force to grip the object, which reduces the amount of energy required to operate them. This can result in significant cost savings over time, as less energy is required to operate the gripper. Compliant microgrippers are also known for their reliability. Because they are designed to be flexible and compliant, they are less likely to break or malfunction than traditional rigid grippers. This makes them ideal for use in applications where reliability is critical, such as in manufacturing processes or medical procedures. In conclusion, compliant microgrippers offer numerous advantages over traditional rigid grippers. They are versatile, precise, safe, efficient, and reliable, making them ideal for a wide range of applications across various industries. As technology advances, we can expect to see even more innovative uses for compliant microgrippers. Microgrippers for tissues or cells that require direct mechanical handling need very complex movements and specific materials because biological cells can be damaged during manipulation [16,17,18]. Manipulating micro-objects has some particularities, such as the influence of adhesion force, material structure, vibration, and so on [19,20]. The authors have developed mini and microgrippers with flexure hinges of different shapes and piezoelectric actuators [21,22].

A similar gripper for biological applications, but with pneumatic control is described in [23]. The mechanism consists of two main parts: the actuation mechanism and the clamping arms. The drive mechanism is a flexible membrane suspended on one end of a tube and applies a force to the base of the clamping device when pneumatic pressure is applied. The fastener consists of two fixed arms and two flexible arms operated by a central platform on which the driving force is applied. To evaluate its performance, five design variables were investigated using finite element analysis: the thickness of the clamping arm, the thickness of the connecting arm, the thickness of the drive screw, the length of the clamping arm, and the angle of inclination of the connecting arm.

A compliant piezoelectric micro-tweezer is a micro-manipulation tool that utilizes piezoelectric materials to produce flexible and compliant motion [24,25,26,27,28,29]. By generating an electrical charge in response to mechanical stress, the piezoelectric materials control the movement of the tweezers [30]. The primary aim of extending the stroke or displacement in a compliant piezoelectric micro-tweezer is to enhance the tool’s precision and range of motion, thereby making it suitable for a broader range of applications, such as manipulating small objects in microfabrication or handling delicate biological specimens.

In recent years, optical tweezers are a type of instrument that use a highly focused laser beam to trap and manipulate microscopic objects, such as in vivo cells and small particles. They have high precision and can manipulate micro-objects with very fine control, making them a preferred option for certain biological applications, but are too expensive [31].

The current study has introduced a novel and improved microgripper design incorporating a compliant mechanism driven by an incremental micro-motor to allow for rough pre-adjustment of the distance between the end-effectors (micro-clamps). The fine adjustment is then carried out using piezoelectric actuators. This innovative approach enables the maximum stroke of a microgripper, typically limited to 300 µm, to be extended up to 2.7 mm with minimal costs and without compromising the pre-tension resolution.

## 2. Design and Modelling of the Whole System

**Figure 1:**
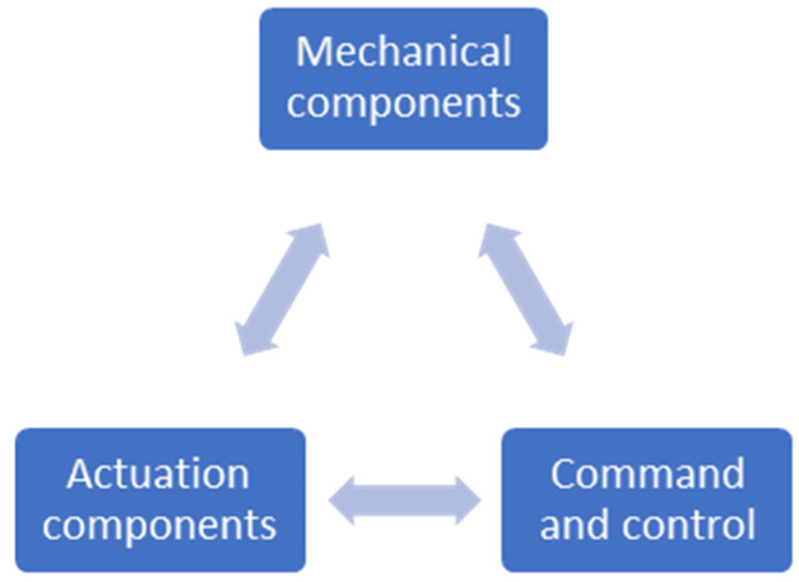
The modules for the mechatronic system.

Design for the proposed micro-tweezers system involves constructive simplicity, with a small number of components, integrated structure, and high reliability, as well as the micrometric displacement and positioning accuracy within a micrometric working range. Figure 1 shows the modules proposed for the micro-tweezers system, including mechanical, actuation, and command and control modules that comprise the mechatronic system.

The mechanical module comprises a compliant mechanism and rigid mechanical components, while the actuation module consists of electromagnetic and piezoelectric components. Additionally, the command-and-control system is designed to support both actuation modes.

**Figure 2:**
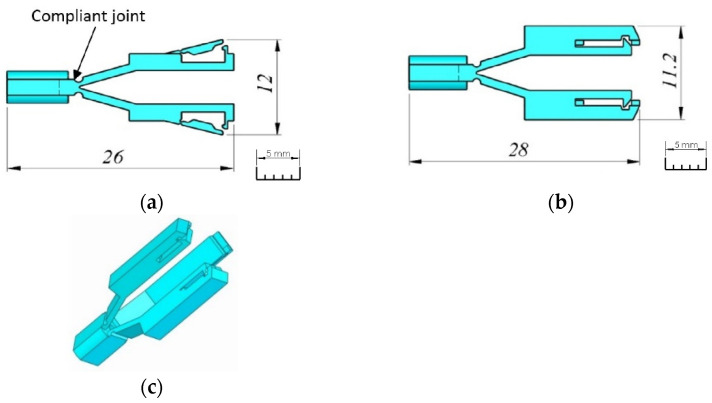
The compliant mechanism (**a**) two arms open; (**b**) two arms closed; and (**c**) three arms closed.

For the design process, we create a 3D model of the compliant mechanism with arms for the tweezers, as shown in Figure 2. The overall dimensions for the compliant mechanism are also considered. The final structure for the gripper will be attached to the arms of the compliant mechanism to form the small tweezers. The compliant mechanism can be designed with two arms with flexure hinges, as shown in Figure 2a,b, or with three arms, as shown in Figure 2c. In Figure 2a, two open arms are visible for attaching the tweezers, while in Figure 2b, the same two arms are shown in the closed position. To ensure safe handling, the gripper is designed with three arms, as shown in Figure 2c.

**Figure 3:**
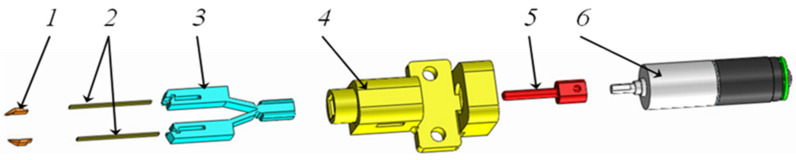
Exploded 3D structure of the system with micro-tweezers.

Figure 3 displays the 3D model of the structural components of the micro-tweezers system. The compliant mechanism actuation mechanical components (3) are integrated into a main component (4), which is connected to the slider component (5), and the compliant mechanism (3) via an elastic joint. The slider component is attached to the stepper motor (6). The tweezers include two parts, which are the piezoelectric bending actuator (2) and the end-effector parts (1), which can have diverse profiles for various microparticles or microcomponents. 

**Figure 4:**
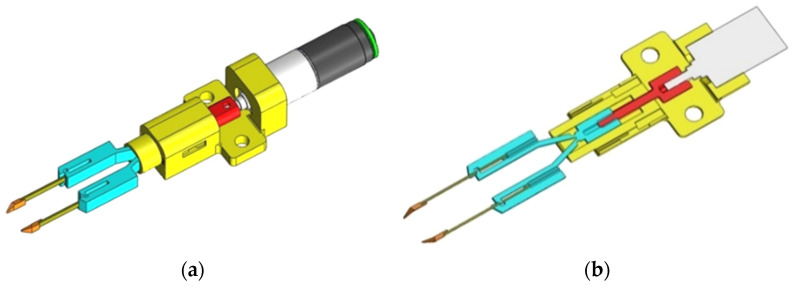
Three-dimensional view of the grip system (**a**); longitudinal section (**b**).

Figure 4 shows an overview of the handling system (Figure 4a) and the longitudinal section (Figure 4b).

**Figure 5:**
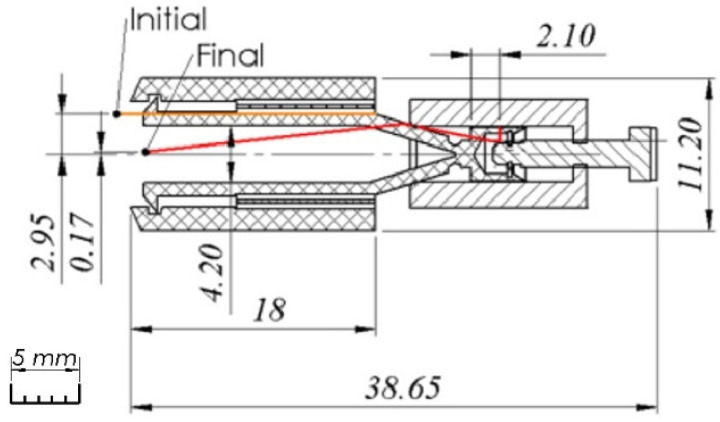
The overall dimensions of the handling system.

The section drawing with the overall dimensions of the handling system without tweezers is presented in Figure 5. Here, it can be observed that the material for the compliant mechanism is non-metallic, and the main component and slider component are metallic.

**Figure 6:**
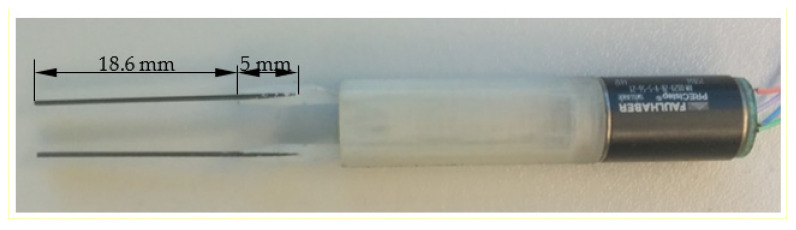
Micro-tweezers system assembled.

The compliant mechanism is a 3D-printed part made of UV-cured resin. The piezo bending components have a total length of 23.6 mm, of which 5 mm have been mounted in the plastic structure of the compliant arms, and the remaining 18.6 mm is the active part (Figure 6). The material used in constructing the piezo elements is a piezoceramic of PSI-5H4E type (the equivalent for PZT-5H) with a thickness of 191.62 μm. The piezoelectric bender comprises a bi-morphic sandwich of two piezoceramic plates with nickel electrodes of opposite polarizations.

Additionally, the end-effectors of the tweezers are mounted on the bending piezo actuators, which can have displacements in both directions.

**Figure 7:**
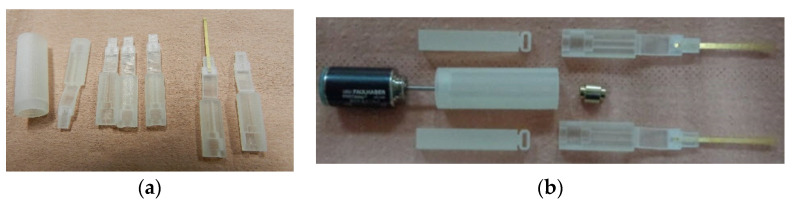

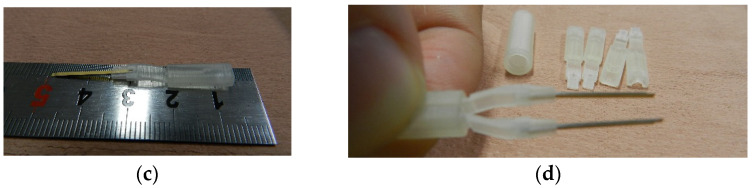
Prototype for the system with micro-tweezers: separate parts for micro-tweezers (**a**), system before assembling (**b**), dimension scale of a tweezer (**c**), assembled system (**d**).

The prototype for the system with micro-tweezers for biomedical applications was manufactured as a compact system, as presented in Figure 7.

Figure 7a presents the components of one arm before being assembled. All the parts, including the stepper motor, actuation mechanical components, main component, slider component, and the compliant mechanism are presented separately in Figure 7b. Figure 7c,d show the scale of one arm and the whole assembly, respectively.

## 3. Kinematic Analysis for the Handling System

**Figure 8:**
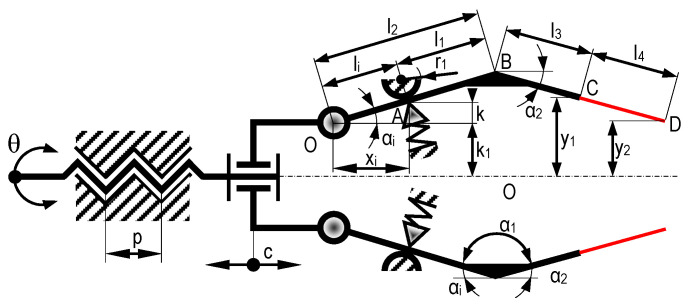
Kinematic scheme of the system with micro-tweezers.

After modeling the system with micro-tweezers, a kinematic analysis of the entire system was conducted. The equivalent kinematic scheme is necessary to support the kinematic calculations, as shown in Figure 8.

Constant values: *k*, *k*_1_, *r*_1_, *α*_0_, *x*_0_, *α*_i_, *l*_2_, *l*_3_, *l*_4_.

*k*—distance between the center of rotation of the flexible joint and the inner wall of the “main component”

*k*_1_—distance between the symmetry axis of the gripper and the rotation center of the flexible joint

*α*_0_—initial angle of the OB element

*x*_0_—initial position of the tweezer

*α_i_*—angle in the different positions

*l*_2_—length of the OB segment of the arm

*l*_3_—length of the BC segment of the arm

*l*_4_—length of the CD segment of the arm

The fixed support, with radius *r*_1__,_ and the opposite elastic support, are fixed in position relative to the system’s base.

For the initial situation (depicted in Figure 6), the initial inclination angle of the OB element is *α*_0_, which is known $\left(tg\alpha_{0}=\frac{k}{x_{0}}\right)$, and *x_i_* = *x*_0_. When the motor shaft stepper is turned step by step, the cylindrical coupling will move to the left, resulting in a stroke denoted by *c*. The parameter *x_i_*, will now be:

(1) $$x_{i}=x_{0}+c$$

Therefore, the angle *α_i_* accordingly, will be:

(2) $$tg\alpha_{i}=\frac{k}{x_{i}}=\frac{k}{x_{0}+c},\;\alpha_{i}=arctg\left(\frac{k}{x_{0}+c}\right)$$

In this situation, the coordinates of point B in a reference system originating from O, shall be:

(3) $$\left\{\begin{array}{c} x_{B}=l_{2}\cos\alpha_{i}=l_{2}\cos\left[arctg\left(\frac{k}{x_{0}+c}\right)\right]\\ y_{B}=l_{2}\sin\alpha_{i}=l_{2}\sin\left[arctg\left(\frac{k}{x_{0}+c}\right)\right] \end{array}\right.$$

On the other hand, the arms AB and BC, being rigidly joined, always have the same relative position between them (*α*_1_ = const.), which means that:

(4) $$y_{C}=y_{B}-l_{3}\sin\alpha_{2}=l_{2}\sin\left[arctg\left(\frac{k}{x_{0}+c}\right)\right]-l_{3}\sin\left[\pi -arctg\left(\frac{k}{x_{0}+c}\right)-\alpha_{1}\right]$$

where:

(5) $$\alpha_{2}=\pi -\alpha_{i}-\alpha_{1}=\left[\pi -arctg\left(\frac{k}{x_{0}+c}\right)-\alpha_{1}\right]$$

In this situation, the distance *y*_1_ can be expressed as:

(6) $$y_{1}=k_{1}+y_{C}=k_{1}+l_{2}\sin\left[arctg\left(\frac{k}{x_{0}+c}\right)\right]-l_{3}\sin\left[\pi -arctg\left(\frac{k}{x_{0}+c}\right)-\alpha_{1}\right]$$

The stroke *c* is achieved by rotating the axis of the stepper motor with an angle *θ*, which results in an axial stroke, according to the relation:

(7) $$c=p\cdot \frac{\theta }{360}$$

where *p* is the step of the threaded axis of the stepper motor.

The angle of rotation of the stepper motor axis can be expressed as a function of the number of ordered steps *n_p_* and the angular step *θ_p_*, as follows:

(8) $$\theta =n_{p}\cdot \theta_{p}$$

The expression for the stroke *c* becomes as follows:

(9) $$c=p\cdot \frac{n_{p}\cdot \theta_{p}}{360}$$

The distance between the compliant elastic elements can now be expressed according to the stroke *c* as follows:

(10) $$2y_{1}=2k_{1}+2l_{2}\sin\left[arctg\left(\frac{k}{x_{0}+p\cdot \frac{n_{p}\cdot \theta_{p}}{360}}\right)\right]-2l_{3}\sin\left[\pi -arctg\left(\frac{k}{x_{0}+c}\right)-\alpha_{1}\right]$$

The expression for the distance between the extremities of the piezo elements in the composition of the microactuator, resulting from the controlled deformation of the compliant elements (without the deformations given by the piezoelectric effect), following the realization of the stroke *c*, due to the rotation of the axis of the stepper motor, is obtained by determining the following successively:

(11) $$y_{D}=y_{B}-\left(l_{3}+l_{4}\right)\sin\alpha_{2}=l_{2}\sin\left[arctg\left(\frac{k}{x_{0}+c}\right)\right]-\left(l_{3}+l_{4}\right)\sin\left[\pi -arctg\left(\frac{k}{x_{0}+c}\right)-\alpha_{1}\right]\;2y_{2}=2k_{1}+2y_{D}=2k_{1}+2l_{2}\sin\left[arctg\left(\frac{k}{x_{0}+p\cdot \frac{n_{p}\cdot \theta_{p}}{360}}\right)\right]-2\left(l_{3}+l_{4}\right)\sin\left[\pi -arctg\left(\frac{k}{x_{0}+c}\right)-\alpha_{1}\right]$$

The mechanical sensitivity, *s_M_*, of the microactuator, corresponding to the minimum achievable mechanical stroke at the end of the piezo elements for a single-step rotation of the motor, is as follows:

(12) $$s_{M}=2k_{1}+2l_{2}\sin\left[arctg\left(\frac{k}{x_{0}+p\cdot \frac{\theta_{p}}{360}}\right)\right]-2\left(l_{3}+l_{4}\right)\sin\left[\pi -arctg\left(\frac{k}{x_{0}+c}-\alpha_{1}\right)\right]$$

## 4. Experiments Set-Up, Procedure, and Results

**Figure 9:**
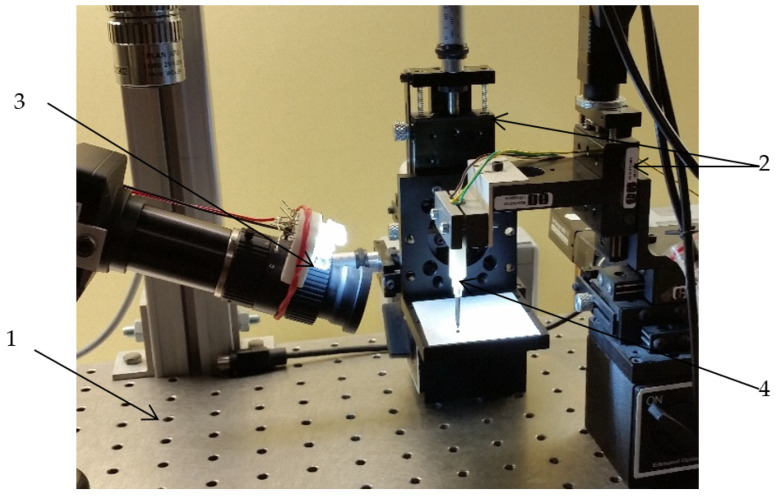
Bench testing for system with micro-tweezers.

To carry out the characterization of the system, an experimental platform (Figure 9) was needed, which includes the following elements:

- The anti-vibration table (1) is made of stainless ferromagnetic steel and weighs 30kg. It is specially constructed to attenuate vibrations.
- The automatic micro-positioning system (2) can make displacements with a pitch of 0.047625 μm and a maximum speed of 8 mm/s. It consists of a mini stepper motor, a microcontroller, and a mechanical system that converts rotational movement into translational motion.
- The video microscope (3) was essential for both the realization of the experiments and for obtaining a series of images captured for static processing. It features an optical system that allows viewing details up to 0.8 μm with internal lighting, a CMOS sensor with a size of 8.6 × 6.9 mm, a speed of 25 frames per second, and FireWire.A interface.
- The system with micro-tweezers (4) for micro-objects and biomedical applications.

The experimental procedure consisted of successive characterization of individual-arm displacements with respect to both stepper motor rotation and piezo-applied voltage, followed by a full micro-manipulation demonstration.

The experimental study focused on the displacement response of the end-effector tweezers, which were actuated by piezo bending actuators, a compliant mechanism, and a stepper motor in response to input signals. 

**Figure 10:**
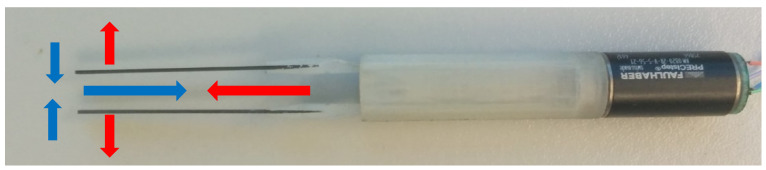
Displacement of the arms. The blue arrow indicates the direction of travel on withdrawal X and the red arrow indicates the direction of travel on X return. Blue arrows indicate the direction of travel on the closing Y while the red arrows indicate the direction of travel on the opening Y.

The movement of the arms was made in the direction of the Y opening, and Y closing, respectively, X withdrawal, and X return as depicted in Figure 10.

**Figure 11:**
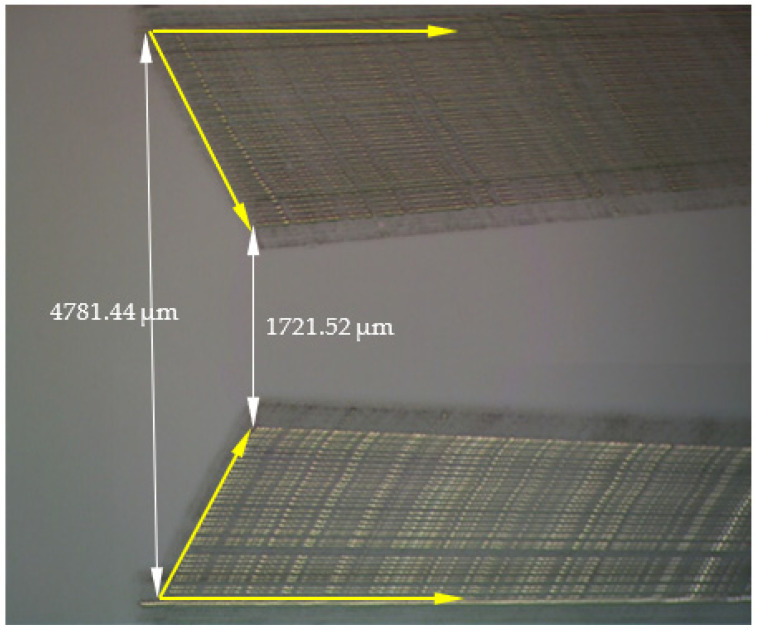
X-Y movement of the arms.

In the Figure 11, it can be seen the movement of the arms in the Y-closing/X-withdrawal direction for both arms. The yellow arrows indicate the direction of movement of the arms.

**Figure 12:**
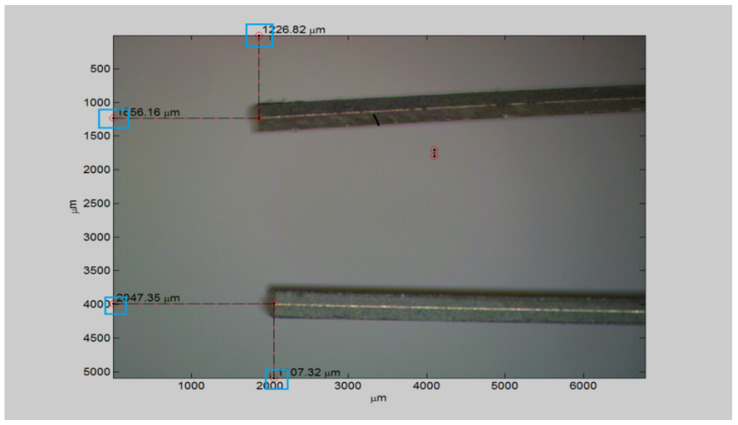
Origin of measurement reference points on x and y directions.

For the measurements on Y and X, it was used as a landmark the top edge of the captured image for the upper arm, for the bottom arm the bottom edge, and for measuring the displacements on the X it was used the left edge (Figure 12).

**Figure 13:**
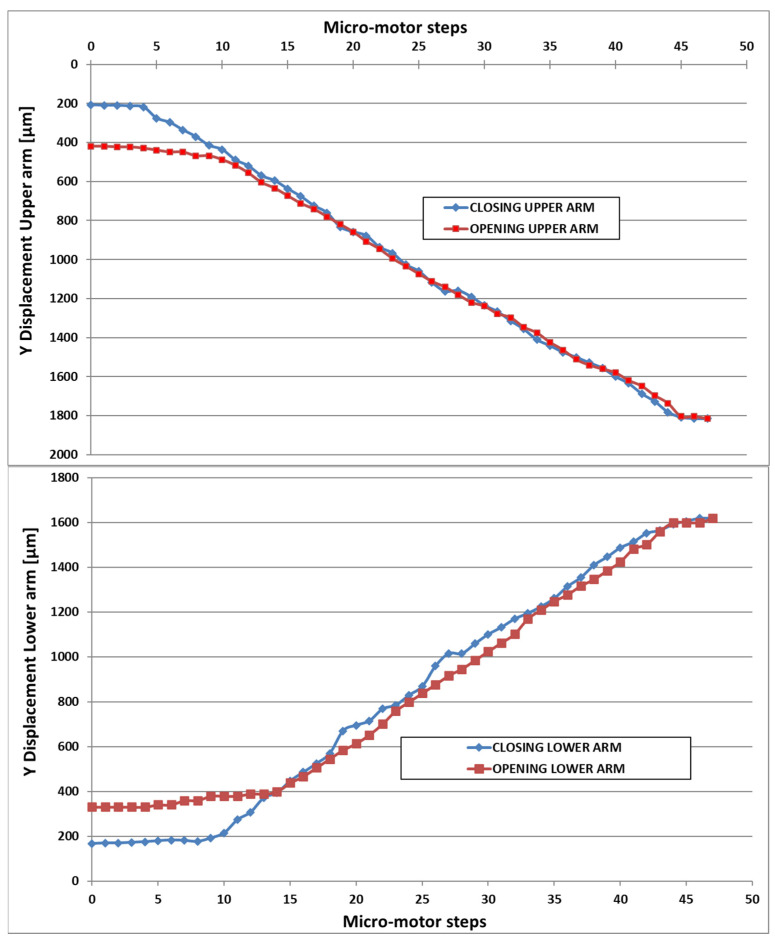
Displacement characteristic in Y-direction for both arms.

The graphs below (Figure 13) plot the closing-opening displacement characteristic in the Y-direction for both arms, showing a millimeter drive range and corresponding linearity.

**Figure 14:**
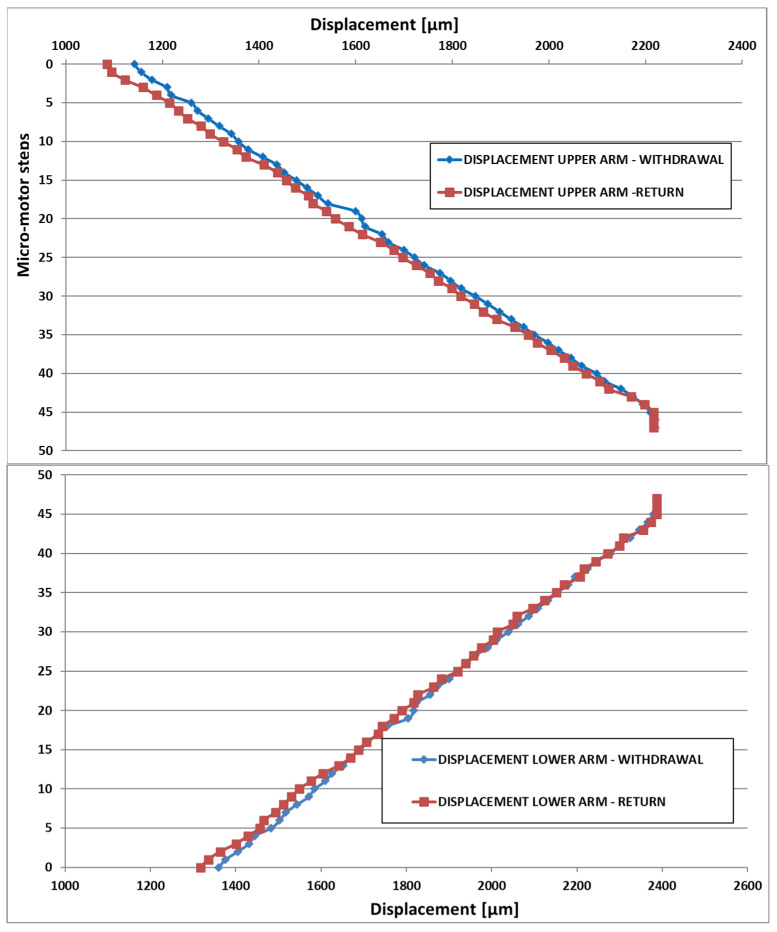
Displacement characteristic in X-direction for both arms.

In the next figure (Figure 14), the displacement characteristic in the X direction is presented for both withdrawal and return movements.

**Figure 15:**
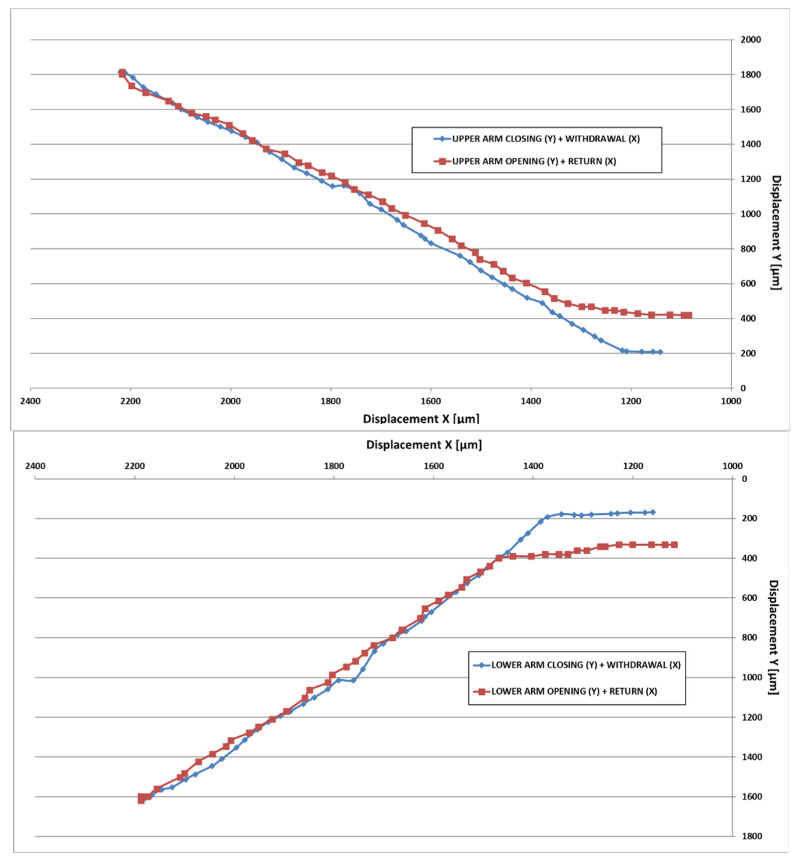
X-Y compound displacement for both arms.

In the graphs below (Figure 15), the compound displacement characteristic between Y + X is plotted.

The maximum distance between the arms is 4781.44 μm and the minimum is 1721.52 μm (see Figure 11).

The positioning resolution on each micromotor step is 40 microns per arm. This gives a total range of 1300 microns per arm. However, the positioning resolution can become submicrometric due to the piezoelectric bender actuator.

According to the sequences presented above, it was possible to manipulate a series of objects with overall dimensions between 10–2700 μm, in a teleoperated way, achieving their precise positioning.

A final measurement test was carried out on the end-effector parts, which are mounted as shown in Figure 3 at the extremity of the piezoelectric bending actuator. These actuators were tested separately by keeping the piezo bender at 0 V.

**Figure 16:**
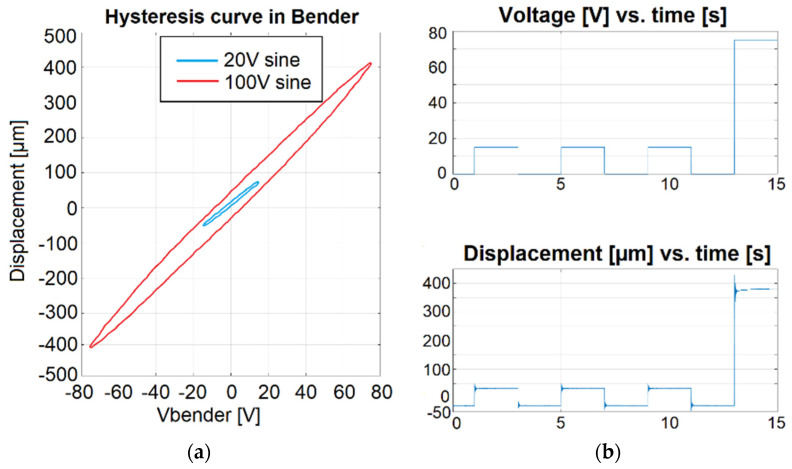
Piezoelectric bender output: (**a**) displacement hysteresis and (**b**) time response.

When a sinusoidal signal with a value of ±75 V was applied to the piezoelectric bender, a 405 µm displacement stroke resulted (Figure 16a). The voltage/displacement values versus time are presented in Figure 16b.

Additional experimental testing was performed with micro-objects manipulated using different shapes of end-effectors, following the methodology developed by the authors of [21,22,23,24]. There were four objects, two of which were identical in size. They were 0.26 mm thick ceramic squares with the following dimensions: 0.26 × 0.26 mm, 0.93 × 0.93 mm, 1.95 × 1.95 mm.

**Figure 17:**
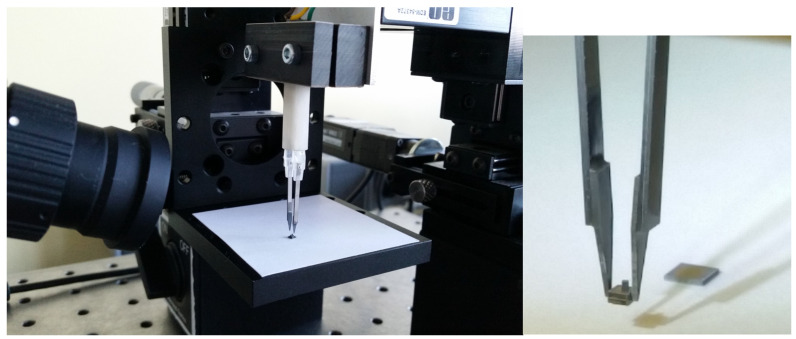
Manipulation of chip-size micro-objects. End-effectors details.

In Figure 17, is presented the bench testing for system with micro-tweezers during the process of positioning of a micro-object.

The widths of the micro-tweezer elements are as follows:

- Piezoceramic plate thickness 191.62 µm
- Electroconductive adhesive thickness ~23.95 µm
- Piezoelectric element thickness 407.19 µm (191.62 × 2 = 383.24 + 23.95 = 407.19)
- Thickness of the end = effector 263.47 µm

**Figure 18:**
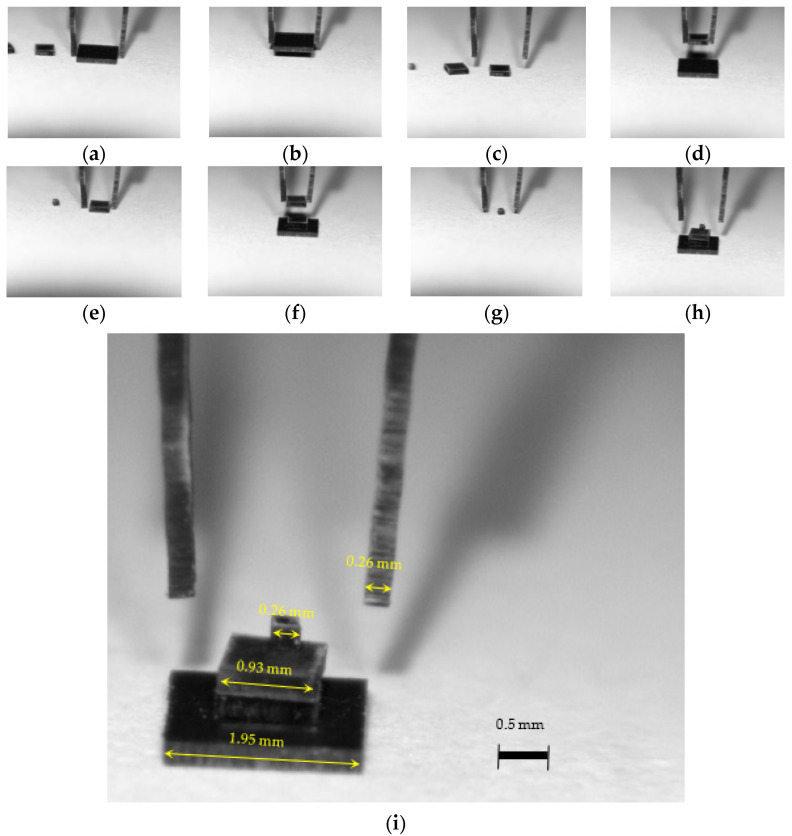
Sequences from micromanipulation operations: prepositioning (**a**), lowering the arms (**b**), clamping (**c**), lifting the micro-object and horizontal displacement (**d**), object positioning (**e**), clamping of the second micro-object (**f**), second micro-object lifting (**g**), second micro-object positioning (**h**) and micro-objects and tweezers dimensions (**i**).

In Figure 18, the sequences of micromanipulation operations can be seen: prepositioning (Figure 18a); lowering the arms (Figure 18b); clamping, lifting the micro-object and horizontal displacement (Figure 18c,d); object positioning (Figure 18e); clamping, micro-object lifting and positioning (Figure 18f–h); and micro-objects dimensions (Figure 18i).

The tweezers can be controlled for the left and right parts separately when 0–150 tension is applied. The piezoelectric bender with ±450 μm displacement and interchangeable end-effector can have a hysteresis of ±12% per the technical description.

## 5. Conclusions

Overall, the goal of this application was to demonstrate the micromanipulation capabilities of the realized mechatronic device. The mechatronic device was designed and made mainly for biomedical applications. However, by obtaining positioning accuracy and a submicrometric resolution, the main framework of micromanipulation was extended to involve “pick and place” operations, the micromanipulation of “rigid” or “soft” objects, and the micro-assembly of parts with complex, three-dimensional geometry.

Using a combined actuation module for both electromagnetic and piezoelectric components, the overall range of the gripper increased while the positioning resolution was maintained.

The advantages of this new and improved microgripper are:

1. Allows for higher accuracy and more precise control over the applied force;
2. The design offers greater flexibility when compared to traditional microgripper designs due to the ability to adjust the end-effectors using piezoelectric actuators;
3. Extended stroke of up to 2.7 mm with minimal costs and without compromising the pretension resolution;
4. High adaptability and versatility to handle a wide range of objects of various shapes and sizes;
5. Robust construction for reliable operation in harsh environments;
6. Safe and efficient operation, even at small-scale operations.

Regarding the choice of a drive system and a manipulation strategy necessary for the proper functioning of a microgripper, the following is noted:

- The material from which a micromanipulator/microgripper is built is also determined by the different environmental conditions in which it operates, such as air, liquids, biological media, clean rooms, and sterile environments.
- The precise grip of objects of different shapes imposes certain conditions on the tightening forces applied by the mechanical structure, which is provided with terminal elements corresponding to the arms.
- Objects manipulated in non-industrial applications, particularly in biological applications, are extremely fragile, necessitating fine control of the clamping force and a reduced gauge comparable to that of manipulated objects.
- The biocompatibility of materials is an important factor in determining the choice of drive for a microgripper.

The microgripper can handle objects of varying shapes and sizes and conform to a wide range of cells or tissues. It is important to note that the benefits of a compliant microgripper are highly dependent on the specific application and context in which it is used. The microgripper has a good working resolution due to the double actuation principle (electromagnetic and piezoelectric) offering degrees of flexibility, adaptability, and safety when working with delicate objects or biological samples.

The optical tweezers, on the other hand, have a large working space, high precision, and can manipulate biological cells with fine control. In terms of comparison, the choice of the gripper depends on the specific application and requirements. It should be on factors such as the type of cells being manipulated, the required level of precision, and the desired level of safety and adaptability.

In terms of cost, compliant grippers are less expensive than optical tweezers which require highly specialized components like lasers and optics. In conclusion, when considering cost and precision, a compliant gripper is less expensive than optical tweezers, while optical tweezers offer the highest level of precision. As with any technology, the choice of the gripper should be based on a careful evaluation of the specific requirements of the application.

One reason why compliant grippers may be safer than other types of microgrippers is that their compliance allows them to exert less force on the object being gripped. Traditional grippers typically made of rigid material like metal may apply too much force on an object, potentially damaging it. In this case, the compliant microgripper can be designed to grip an object with just enough force to hold it securely without exerting an excess force that could cause damage.

At this small scale, the compliant grippers show obvious advantages over the classic hinge structures: easier miniaturization, reduced complexity, compatibility with 3D printing, zero backlash, and improved linearity.

## Data Availability

The data presented in this study are available on request from the corresponding author. The data are not publicly available due to privacy.
